# Evidence on Hidradenitis Suppurativa as an Autoinflammatory Skin Disease

**DOI:** 10.3390/jcm13175211

**Published:** 2024-09-02

**Authors:** Martina D’Onghia, Dalma Malvaso, Giulia Galluccio, Flaminia Antonelli, Giulia Coscarella, Pietro Rubegni, Ketty Peris, Laura Calabrese

**Affiliations:** 1 Dermatology Unit, Department of Medical, Surgical and Neurological Sciences, University of Siena, 53100 Siena, Italy; martina.donghia@gmail.com (M.D.); giulia.galluccio95@gmail.com (G.G.); pietro.rubegni@unisi.it (P.R.); 2 UOC di Dermatologia, Dipartimento di Scienze Mediche e Chirurgiche, Fondazione Policlinico Universitario A. Gemelli - IRCCS, 00168 Rome, Italy; malvasodalma@gmail.com (D.M.); ketty.peris@unicatt.it (K.P.); 3 Dermatologia, Dipartimento di Medicina e Chirurgia Traslazionale, Università Cattolica del Sacro Cuore, 00168 Rome, Italy

**Keywords:** hidradenitis suppurativa, autoinflammation, interleukin-1 family, inflammasome

## Abstract

Hidradenitis suppurativa (HS) is a chronic and debilitating inflammatory skin disease that often exhibits heterogeneity in its clinical presentation, especially in the context of its rare syndromic forms. The pathogenesis of HS results from a complex interplay of genetic predisposition, innate and adaptive immunity dysregulation, smoking, obesity and environmental factors. In the early phase of the disease, the innate immune system is hyperactivated, contributing to tissue damage and triggering the activation and amplification of the adaptive immune response, which plays a pivotal role in the chronic stages of the disease. Recent studies focused on elucidating the importance of innate immunity impairment and autoinflammation in HS and increasing evidence has emerged on the occurrence of the disease in the context of well-known monogenic and polygenic autoinflammatory syndromes (AIDs). This review provides a comprehensive examination of the current scientific background supporting the contribution of autoinflammation to HS etiology, including genetic data, molecular studies and clinical evidence, as well as the association between HS and AIDs. However, further research is needed to shed light on the pathogenic mechanism of this challenging condition and to identify potential perspectives for future therapeutic approaches.

## 1. Introduction

Hidradenitis suppurativa (HS), also known as acne inversa or Verneuil disease, is a chronic and debilitating inflammatory skin condition typically involving the hair follicle of apocrine gland-bearing skin [1,2]. It usually appears after puberty, especially during the third decade [3], with a typical recurrent and progressive course, which leads to a profound impact on the patient’s quality of life [4].

Depending on the severity and the stage of the disease, patients with HS may present with a variety of skin lesions, ranging from papules and pustules, comedones and inflamed cysts to recurrent painful, deep-seated nodules and abscesses, which may develop into draining sinus tracts and hypertrophic scars [5]. 

Lesions typically appear in the intertriginous areas, with a preferential distribution on the axillary, inguinal and submammary folds in females, whereas the perianal area and buttocks are more commonly involved in males [6,7,8]. In addition, patients may experience malodorous discharge, disfigurement, itching and pain [9], with a significant emotional impact and social isolation due to fear of stigmatization [10,11]. The global prevalence of HS ranges from 0.00033 to 4.1% (most likely 0.7–1.2% in the European and US populations) [5]. Despite the high burden of the disease on patients, the therapeutic options for HS are currently limited. Adalimumab (anti-TNF-α monoclonal antibody(mAb)) [12], secukinumab ((mAb targeting interleukin(IL)-17A) [13] and bimekizumab (mAb selectively inhibiting both IL-17A and IL-17F) [14] are the only approved biologic agents for the treatment of moderate-to-severe HS and provide a clinical benefit in approximately 40% of HS patients, still resulting in a high unmet clinical need. 

The pathogenesis of HS is largely unknown, resulting from a complex interplay of genetic predisposition, smoking, environmental factors and innate and adaptive immune dysfunction [15]. Increasingly, studies have supported the primary role of innate immunity dysregulation in HS, and thus of autoinflammation. Autoinflammation involves a predominant dysregulation of innate immune responses, in some cases caused by an overactivation of the inflammasome, a multiprotein cytosolic complex responsible for sensing cellular stressors [16]. Also, the primary role of innate immunity dysregulation and autoinflammation in HS is supported by its presence in the context of autoinflammatory diseases (AIDs), such as PASH (pyoderma gangrenosum (PG), acne and HS) and PAPASH (pyogenic arthritis, acne, PG and HS) syndromes [17]. Regarding this background, understanding the pathogenetic mechanisms of HS could shed light on new and unexplored molecular pathways also shared by AIDs conditions and pave the way for new targeted therapeutic strategies. 

Finally, according to the current literature and ongoing research, HS should be regarded as a potential subtype of autoinflammatory keratinization disease (AiKD), a recent disease entity proposed by Akiyama et al. [18]. AiKDs are characterized by the following three criteria: (i) the primary sites of inflammation are the epidermis and the upper dermis; (ii) inflammation leads to hyperkeratosis, which is the main and characteristic phenotype of AiKDs; and (iii) AiKDs have primary genetic causative factors associated with the hyperactivation of innate immunity (autoinflammation), mainly in the epidermis and upper dermis [19].

Thus, the role of this review is to thoroughly explore the current scientific evidence underlying the role of autoinflammation in HS, ranging from genetic data to molecular studies and clinical evidence, as well as to provide a forward-looking update on the relationship between HS and AIDs. 

## 2. Pathogenesis of Hidradenitis Suppurativa

For years, HS has been considered a bacterial skin infection of the terminal follicle, resulting in an intense and destructive local inflammatory response due to its typical clinical features, such as purulent discharge in apocrine gland-bearing areas and clinical benefit with antimicrobial treatment [20]. However, microbiologic screening of purulent discharge usually reveals negative cultures or a mixture of normal flora and skin commensals, with no specific growth of bacterial agents in culture to date [21]. The role of bacteria remains controversial as it has been found that the microbiome in HS lesional and non-lesional skin differs from healthy controls, supporting the hypothesis of a link between a dysbiotic cutaneous microbiome and HS lesions [22].

Although biopsies are not routinely performed for diagnostic purposes, the histopathologic examination of lesions is certainly helpful in understanding the events underlying the pathogenesis of HS: typical histological patterns are hyperkeratosis of terminal follicular openings, hyperplasia of the follicular epithelium and perifolliculitis with immune cell infiltration [23].

**Figure 1:**
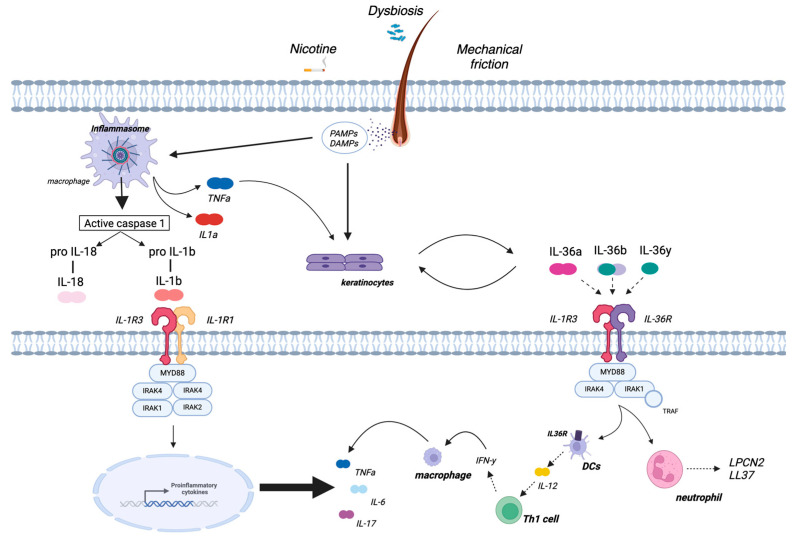
Pathogenesis of HS. Currently accepted paradigm of HS pathogenesis assumes an initial involvement of the terminal Pilosebaceous Unit (PSU) leading to surrounding inflammation through a multistep process that includes follicular occlusion and subsequent rupture. This results in the release of Pathogen-Associated Molecular Patterns (PAMPs) and Damage-Associated Molecular Patterns (DAMPs), with consequent activation of the innate immune response. The inactive precursor of IL-1β (pro-IL-1β) is converted into the active form IL-1β through the inflammasome. Activated keratinocytes produce IL-36 cytokines, which further promote the production of proinflammatory cytokines, such as IL-12 and IL-23, which, in turn, selectively trigger adaptive immunity in a feed-forward loop.

A key pathogenic event in HS is now considered to be the hyperkeratotic plugging of the terminal hair follicle opening, with consequent occlusion and dilatation/elongation of the hair follicle, leading to rupture of the follicular epithelial wall and bacterial biofilm formation [21]. These events trigger the immune response and result in the release of Damage and Pathogen Associated Molecular Patterns (DAMPs and PAMPs, respectively) and consequent activation of innate immunity. In detail, resident immune cells, such as macrophages, are hyper-responsive to PAMP stimulation in HS due to an increased expression of toll-like receptor 2 (TLR2) and secrete pro-inflammatory cytokines, including TNFα, IL-6 and IL-1β [24]. The inactive precursor of IL-1β (pro-IL-1β) is converted into the active form IL-1β by a cleavage process mediated by a multiprotein complex called the inflammasome [25]. Therefore, over-activation of the innate immune system appears to be central, especially in the early phase of the disease, due to the massive production of inflammatory cytokines in response to dermal seeding of keratin, sebum, bacterial components and cellular debris [26]. Indeed, according to several studies, one of the most prominent immunological features observed in HS-injured skin is the upregulation of TNF-α and IL-1β pathways, mainly secreted by macrophages [23,27,28,29]. These cytokines induce the expression of a broad range of chemokines, including CXCL8, CXCL11, CCL2 and CCL20 in keratinocytes and CXCL1 and CXCL6 in fibroblasts, bolstering further infiltration of immune cells [27]. This process includes granulocytes, T cells, B cells and monocytes, which locally differentiate into macrophages and dendritic cells, and enhances the fibroblastic secretion of matrix metalloproteinases (MMP), which are involved in tissue destruction and tunnel formation [27]. In addition, activated keratinocytes release pro-inflammatory cytokines such as IL-1β, IL-36α, IL-36β and IL-36γ (all of the IL-1 cytokine family) [30]. These cytokines target different cell types, from keratinocytes to dendritic cells, and promote the production of pro-inflammatory cytokines such as IL-12 and IL-23, which induce adaptive immunity (Th1 and Th17 responses) [31]. Dendritic cells also produce IL-36, creating an autocrine loop that further amplifies inflammation [30]. In the chronic phase of HS, an imbalance between Th17 cells and regulatory T cells (Tregs) is found, with elevated serum levels of IL-17 and a local increase in the ratio of proinflammatory Th17 cells and Tregs [32]. The enrichment of Th17 cells in HS skin and the dysregulation of the Th17:Treg cell axis are likely driven largely by increased IL-1β and IL-6 because of innate immunity overactivation [33]. A schematic representation of HS pathogenesis is provided in Figure 1. 

## 3. Preclinical Evidence on the Role of Autoinflammation in Hidradenitis Suppurativa

To date, there is a wealth of preclinical scientific evidence, ranging from genetic to molecular, underscoring the role of autoinflammation in HS [34].

The importance of genetic factors in HS is well known and established, with approximately 35–40% of patients having a family history of HS [35,36].

In 2010, heterozygous loss-of-function mutations in NCSTN, PSENEN and PSEN1 were identified in six Chinese patients with HS [37]. These genes are known to encode for components of γ-secretase, a multi-subunit complex involved in the signal transduction associated with NOTCH receptors, which regulate both proliferation and differentiation of keratinocytes [38]. 

Interestingly, not only are genes involved in keratinization processes implicated in HS and its syndromic forms but also those related to autoinflammation [39]. Specifically, genes associated with HS can be categorized into two distinct groups. The first group includes NCSTN, PSENEN, PSEN1, POFUT1, POGLUT1, KRT17, KRT6A, FGFR2 and GJB2. Mutations in these genes result in autoinflammation, which is preceded by keratinization. It has been proposed that this HS subtype could be regarded as an AiKD in the broad sense. The second group of genes includes MEFV, NOD2, LPIN2, NLRP3, NLRP12, PSMB8, MVK, IL1RN and PSTPIP1, whose mutations lead to keratinization preceded by autoinflammation. Conversely, this HS subtype could be regarded as an AiKD in sensus stricto, as it is most likely the autoinflammation that is at the heart of the pathogenic process [39].

In detail, the PSTPIP1 gene encodes the enzyme proline–serine–threonine phosphatase-interacting protein 1 (PSTPIP1) [40]; mutations that cause its overactivity lead to its hyperphosphorylation and increased assembly of the pyrin inflammasome, ultimately resulting in the uncontrolled release of IL-1β [40]. Additionally, the MEFV gene encodes for pyrin, a clue component of the pyrin inflammasome [41]. HS may affect patients with familial Mediterranean fever (FMF), a prototypic monogenic AID, carrying *MEFV* mutations, with a higher frequency of MEFV mutations reported in HS patients than in healthy controls, suggesting a potential contribution to HS pathogenesis [42,43].

Furthermore, mutations in MEFV, NLRP3, NLRP12, NOD2 and LPIN2 have been investigated in genomic DNA samples from patients affected by pyoderma gangrenosum (PG) and its syndromic variants, including also HS in their spectrum, which will be discussed in greater detail later in this review [44].

Based on genetic evidence confirming the autoinflammatory nature of HS, several experimental studies have thoroughly investigated the role of the IL-1 family, a key element of innate immunity, in HS [45,46].

Specifically, gene expression analysis of immune cells isolated from HS skin revealed an overexpression of B cells, antimicrobial peptides and IL-17A, along with enrichment of IL-1-related pathways and IL-17A-producing Th17 cells in HS skin compared to healthy skin [47]. In another study, expression levels of IL-1 pathway mediators were quantified in lesional HS skin samples in comparison to healthy donor skin samples, using real-time PCR (RT-qPCR) [27]. IL-1β was the most expressed cytokine in HS lesions compared to healthy skin, and its expression was even more pronounced than in other inflammatory skin diseases such as psoriasis. Moreover, IL-1β levels were found to be elevated in perilesional HS skin [27].

As IL-1β is processed by caspase-1, Kelly et al. [28] employed flow cytometry using the caspase-1 fluorochrome inhibitor of caspases (FLICA) to detect active caspase-1 in skin biopsies. The results showed that caspase-1 activation was enhanced in live cells from HS lesional skin compared to cells from perilesional skin. Furthermore, caspase-1 inhibition was found to partly suppress IL-1β secretion in HS lesional skin, indicating a high expression of caspase-1 in HS lesions [28]. 

Not only IL-1 but also IL-36 cytokines have been studied in HS. The IL-36 subfamily members belong to the IL-1 family and have been demonstrated to play a role in HS pathogenesis [48]. In 2017, skin biopsies were collected from the affected sites of HS patients for in vivo mRNA investigation of IL-36α, IL-36β, IL-36γ and IL36Ra by RT-PCR. Increased mRNA expression of IL-36α, IL-36β and IL-36γ was found in the lesional skin of HS patients as well as in patients affected by acne, suggesting a common pathogenesis of the two skin conditions. Conversely, no increase in IL-36Ra, which is an anti-inflammatory member of the IL-36 subfamily, was detected. Furthermore, the expression of IL-8 was investigated in skin conditions such as acne, HS and psoriasis. A significant increase was observed in all settings, with the highest level of IL-8 expression observed in HS and the lowest in psoriasis [48]. These results were corroborated by another study, performing immunostaining on skin biopsies of inflammatory HS lesions. The expression levels of IL-36α, 36β and 36γ were all significantly higher in lesional and perilesional HS skin than in healthy controls. Furthermore, immunohistochemical analysis revealed a primary expression of IL-36 cytokines by keratinocytes, highlighting a pivotal role of the IL-36 family in the crosstalk between keratinocytes and immune cell activation and, in turn, in determining the outbreak of HS lesions [49]. Finally, systemic levels of IL-36 were investigated in healthy donors, HS and psoriatic patients’ serum through ELISA. Patients affected by HS exhibited elevated levels of the isoforms IL-36α, IL-36β and IL-36γ, with no detection of IL-36RA [50].

Recently, Moran et al. [51] employed single-cell RNA sequencing (scRNA-Seq) to investigate CD45+ cells isolated from healthy control skin and HS surgical resection tissue. Myeloid clusters in HS skin showed significantly higher levels of NLRP3, IL-1β and IL-18 in HS samples compared to the controls. It is noteworthy that a correlation between IL-1β and Th17-mediated inflammation was revealed, thereby confirming the role of IL-1 in downstream inflammation in the pathogenesis of HS [51].

From a mechanistic perspective, certain monogenic AIDs result in the sustained activation of the NLRP3 or pyrin inflammasomes, which subsequently activate caspase-1, leading to the release of IL-1 and the onset of autoinflammation. Indeed, alongside an increase in IL-1β, elevated levels of caspase-1, NLRP3, IL-6 and IL-18 have been reported in the lesional skin of HS patients [51,52].

Interestingly, an ex vivo explant model was recently used to culture skin in the presence or absence of the prototypical NLRP3 inflammasome inhibitor MCC950. Administration of MCC950 determined a consistent decrease in the levels of IL-1β, TNF-α, IL-17A, IFN-γ, CCL20, CXCL1, IL-8 and IL-36γ from involved HS skin, with no significant change in the release of IL-1α, IL-18, IL-17C, MMP3 or S100A8. This confirms the pivotal role of the NLRP3 inflammasome in shaping the inflammatory burden in HS-affected skin as well as the potential of its blocking in HS treatment [52].

## 4. Clinical Evidence of the Role of Autoinflammation in Hidradenitis Suppurativa 

A primary role for innate immune dysfunction in HS is supported not only by preclinical and genetic data but also by clinical evidence.

### 4.1. Syndromic Hidradenitis Suppurativa 

**Figure 2:**
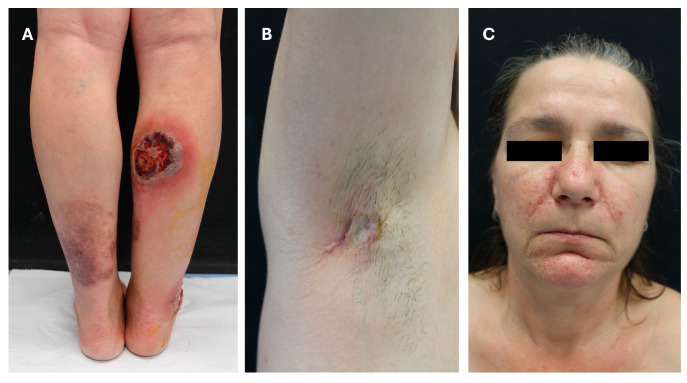
Syndromic HS – PASH syndrome. PASH syndrome is a rare autoinflammatory syndrome consisting of neutrophil-mediated inflammation, whose dermatological manifestations include the triad pyoderma gangrenosum (PG), acne and hidradenitis suppurativa (HS). The patient exhibited a painful ulcer with violaceous borders, involving the right lower extremity, consisting of PG (**A**). HS manifested with sinus tracts, deep-seated inflammatory nodules, as well as atrophic and rope-like scars, distributed throughout the axillae, inguinal–crural folds and genital area (**B**). Furthermore, the presence of papules, pustules and scarring on the face contributed to the complexity of the clinical presentation of this challenging case (**C**).

It is well documented that HS can occur in the context of complex auto-inflammatory syndromes caused by mutations in pattern recognition receptor (PRP) signaling pathways, as in the case of the auto-inflammatory diseases PASH (PG, acne and HS) and PAPASH (PG, acne, pyogenic arthritis and HS), which are associated with multiple neutrophilic dermatoses [53]. Representative pictures of PASH syndrome are shown in Figure 2.

More recently, psoriatic arthritis (PsA), PG, acne, HS and ankylosing spondylitis (PASS); PG, acne and HS (PsAPASH); pustular psoriasis, arthritis, PG, synovitis, acne and HS (PsAPSASH); PsA, PG, HS and Crohn’s disease (PsAPSC) and vasculitis with PASH (VPASH) were added to the spectrum of syndromic HS [54].

HS often exhibits significant heterogeneity in clinical presentation, complicating the diagnostic assessment of its rare syndromic forms. As a result, diagnosis remains challenging for physicians, resulting from the integration of clinical observation, HS-specific diagnostic criteria, histopathology of lesional skin and laboratory data [55]. In this context, syndromic HS typically experiences more severe forms of the disease, with unusual skin locations and signs of systemic inflammation, showing resistance to conventional treatments.

Despite the fact that PASH syndrome pathophysiology is still unclear, mutations in the coding region of PSTPIP1 have been identified [56], and an increased number of CCTG repeats in the PSTPIP1 promoter region appears to be a consistent finding in PASH patients, governing a complex network of interactions between innate and adaptive immunity and environmental factors [54]. Indeed, it has been shown that CCTG replications are likely to deregulate PSTPIP1 expression, predisposing to neutrophilic skin inflammation via inflammasome-dependent IL-1β production [44]. As longer repeats have also been reported in patients with more severe disease, it is intuitive to speculate that repeat length may have an impact on the severity of the HS phenotype [57]. 

Finally, additional genes involved in other auto-inflammatory diseases, such as NOD2, MEFV, NLRP3 and TNFRSF1A, were investigated in the pathogenesis of PASH syndrome, but no major mutations have been identified [58]. 

The cutaneous manifestations of PASH syndrome may be accompanied by either pyogenic arthritis or PsA in less well-described syndromes named PAPASH [59] or PsAPASH, respectively [60,61]. Interestingly, although a PSTPIP1 mutation has been identified in PAPASH [62], there is currently no evidence for its role in PsAPASH. 

In addition to the above, another recently described syndrome associated with HS is PASS, which combines PG, acne, HS and seronegative spondylarthritis with high fever and severe back pain during the disease flare [63]. During the disease exacerbation, episodes of fever accompanied by elevated serum levels of IL-1β were observed, further substantiating the autoinflammatory nature of this disease [64]. In contrast to other autoinflammatory diseases, such as PAPA and PAPASH, the gene PSTPIP1 was not found to be mutated in PASS [63]. This suggests the possibility of additional mutations in the IL-1 pathway and the necessity for further investigation [64]. 

It is noteworthy that HS is seldom associated with synovitis, acne, pustulosis, hyperostosis and osteitis (SAPHO) syndrome, a clinical entity that affects both the musculoskeletal and dermatological systems [54]. The pathogenesis of SAPHO remains poorly understood. However, it is believed that cytokines, including IL-1β, IL-8, IL-17 and TNFα, play a significant role [65,66]. Specifically, SAPHO syndrome partly depends on the genetically encoded overproduction of IL-1β [67]. More recently, a heterozygous frameshift mutation of NCSTN has been found in one patient affected by the SAPHO syndrome, suggesting a potential genetic link between the two conditions [68]. However, further research will offer important clues by identifying specific genes and pathways, which may further explain the complex pathogenesis of SAPHO syndrome and the potential association between those rare diseases. A list of main syndromic forms of HS is provided in Table 1.

### 4.2. Hidradenitis Suppurativa and Monogenic Autoinflammatory Diseases

The occurrence of HS in the context of monogenic AIDs, primarily FMF, has been recently investigated with the objective of elucidating the etiology of the disease [42]. 

A cohort study conducted on 119 HS patients indicated a potential association between HS and FMF [43]. Two groups were considered: those with complex HS and non-complex HS phenotypes. Complex HS patients were defined as those in Hurley stage III, or those with Hurley stage II and one or more inflammatory diseases, including PG, dissecting cellulitis of the scalp (DCS), arthritis, inflammatory bowel disease or acne conglobata. Overall, five patients (4.2%) were diagnosed with FMF, with a childhood onset and typical symptoms. The prevalence of FMF was higher in the complex HS phenotype (*p* = 0.035), with four FMF patients having an additional inflammatory condition besides HS. Furthermore, the age of onset for HS was lower in FMF patients than in carriers of MEFV variants (19.2 years). The pathogenic mutations of MEFV genes identified in the five FMF-affected patients were as follows: 70% for M694V, 20% for V726A and 10% for E148Q. This study outlined a high odds ratio for FMF diagnosis in the HS population compared to the general Turkish population (OR = 45 CI:16.50-99.84 *p* < 0.001) [43]. In the complex HS group, the allelic frequency of pathogenic MEFV mutations was found to be 23.8%, while in the healthy cohort, it was 11.2%. MEFV mutation carriers exhibited more severe HS than other participants. This observation suggests the potential induction of a proinflammatory state in mutation carriers, which could lead to severe cutaneous disease [43].

Similar results were obtained by Hodak et al. based on the database of Clalit Health Services (CHS), the largest healthcare organization in Israel. The study included 4417 patients with HS and 22,085 controls. FMF was reported in 33 HS patients (0.7%) and in 15 (0.1%) control subjects. This pointed out a marked association between HS and FMF, which was evident in this population both in univariate (OR 11.1; 95% CI 6.0–20.4) and multivariate analysis [69]. An alternative recruitment strategy was employed by Abbara et al., which included 151 patients with FMF from the French adult reference center for FMF. Among the participants, six cases of HS were identified. The resulting HS prevalence in this group was 4%, which corresponds to the upper percentage of HS prevalence in the general population (1–4%). Furthermore, HS patients in the FMF population did not exhibit distinctive clinical characteristics or a different age of onset when compared with the remainder of the cohort. No temporal correlation was observed between the exacerbation of HS and FMF. Consequently, no compelling links between the two diseases were identified in this study [70].

Cases of HS associated with other monogenic AIDs, namely hyper-IgD syndrome (HIDS), have also been reported [71]. HIDS is a genetic disease that arises from mutations in the mevalonate kinase (*MVK*) gene. This gene encodes an enzyme that plays a role in the synthesis of cholesterol and isoprenoids, which are essential for the synthesis of steroids, vitamins, hormones and other important compounds [71]. The reduction of isoprenoids, which are essential for the attenuation of the inflammatory response, is a direct consequence of *MVK* deficiency. Interestingly, it has been demonstrated that an impaired *MVK* activity promotes both hyper-keratinization [72] in the skin and the hyperactivation of pyrin inflammasome with subsequent overproduction of pro-inflammatory mediators such as IL-1β [73]. The clinical presentation of the condition occurs in childhood with recurrent episodes of fever, rash, abdominal pain, oral ulcers and adenopathy, with an average duration of one week [74].

In both cases reported by Guillem et al., the patients were lean and non-smokers, suggesting that environmental factors had a limited impact on the pathogenesis of HS in these subjects. Moreover, response to standard antibiotic and surgical HS therapy was limited, which could be due to a strong inflammation supported by genetic mechanisms. Anti-TNF-α therapy, administered alone or in combination with canakinumab (monoclonal antibody anti-IL-1β), was found to be an effective treatment for HS symptoms, further substantiating the role of these cytokines in the disease pathogenesis [71]. Additionally, a case of concomitant HIDS and HS was reported in 2023, which was also effectively managed by anti-TNFα drugs [75].

A case of paradoxical HS was described as an adverse event occurring during canakinumab administration in a patient affected by MVK deficiency. In this context, it has been theorized that the blockade of IL-1β could have prevented the IL-1β-mediated hair follicle regeneration and re-epithelization induced by Staphylococcus, acting as a trigger for HS development [76]. Furthermore, the lack of MVK activity leads to hyperkeratinization, which could represent another factor promoting HS development in HIDS [72]. However, fewer than 200 HIDS cases have been described to date, making it unclear whether there may be a pathogenic connection between the two diseases or whether the association is coincidental.

### 4.3. Therapeutic Agents Targeting IL-1 Family Pathways 

The link between HS and autoinflammation has led to the development of therapeutic agents aimed at targeting IL-1 family pathways at multiple levels to treat this disease. Indeed, to date, many targeted therapies are in the pipeline to assess the clinical efficacy of IL-1 therapeutic antagonism. Among these is anakinra, a recombinant IL-1R antagonist that inhibits both IL-1α and IL-1β, which has demonstrated comparatively positive outcomes in HS in both a short open-label trial and a randomized clinical trial [77,78]. Other compounds under investigation include bermekimab, a human anti-IL-1α mAb; canakinumab, an mAb that targets IL-1β; and lutikizumab, which potently neutralizes both IL-1α and IL-1β [79]. 

The inhibition of IRAK4 kinases, involved in IL-1 intracellular signaling and therefore potential therapeutic targets in autoinflammatory diseases, is another mechanism of action currently under investigation in HS [80]. Two small molecules, KT-474 and zimlovisertib, which target IRAK4, are under investigation, but preliminary efficacy data are not encouraging, with only 34% of HS patients receiving zimlovisertib in the phase II trial achieving HiSCR50 at week 16 [81].

Other members of the IL-1 family and specifically the IL-36 cytokines have been implicated in the pathogenesis of inflammatory cutaneous diseases, including psoriasis and HS [30]. As a result, the IL-36/IL-36R axis has been proposed as a prospective therapeutic target and certain drugs targeting IL-36 (spesolimab, imsidolimab) are under investigation in HS [34,82]. In detail, a recent proof of concept study of spesolimab in HS showed promising results that need to be confirmed in phase III trials [82].

## 5. Conclusions

Recent research has unveiled the role of dysregulation of innate immunity and the IL-1 family pathway as central mediators in monogenic AIDs, but also in common polygenic disorders, including HS. The role of innate immunity and autoinflammation in HS was first highlighted by genetic evidence and experimental studies. Later on, this concept was further supported by clinical evidence of the occurrence of HS in the context of complex autoinflammatory syndromes linked to mutations in PRR signaling pathways. One such syndrome is PASH, a rare disease that associates multiple neutrophilic dermatoses, including pyoderma gangrenosum, acne and HS. Nevertheless, HS is still a thriving area of current scientific research and remains a major unmet therapeutic need. Additional investigational and preclinical data will expand the treatment options for this challenging and multifaceted disease.

## Figures and Tables

**Table 1:** List of major polygenic autoinflammatory syndromes with HS as a clinical manifestation.

Disease	Clinical Features	Involved Genes (Inheritance Pattern)	Encoded Protein	Function
PASH syndrome	Pyoderma gangrenosum, acne, hidradenitis suppurativa	*NCSTN* *IL1RN* *PSTPIP1* *MEFV* *NOD2* *NLRP3* *PSMB8* *PSEN1/PSEN2*	Nicastrin IL1RA PSTPIP1 Pyrin NOD2 NLRP3 PSMB8	γ-secretase, proteolysis IL-1 pathway Inflammasome, cytoskeleton regulation Inflammasome Inflammasome, NF-κB pathway Inflammasome Proteasome complex
PAPASH syndrome	Pyogenic arthritis, Pyoderma gangrenosum, acne, hidradenitis suppurativa	*PSTPIP1*	PSTPIP1	Inflammasome, cytoskeleton regulation
PASS syndrome	Pyoderma gangrenosum, acne, Hidradenitis suppurativa, ankylosing spondylitis	Unknown	//	//
PsAPASH syndrome	Psoriatic arthritis, pyoderma gangrenosum, acne, hidradenitis suppurativa	Unknown	//	//
PsAPSASH syndrome	Pustular psoriasis, arthritis, pyoderma gangrenosum, synovitis, acne and hidradenitis suppurativa	Unknown	//	//
SAPHO syndrome	Synovitis, acne, pustulosis, hyperostosis, osteitis	*LPIN2* *NOD2* *PSTPIP2* *IL1RN*	Lipin 2 NOD2 PSTPIP1 IL1RA	Lipid biosynthesis pathway Inflammasome, NF-κB pathway Inflammasome, cytoskeleton regulation IL-1 pathway

Legend: Clinical features and genetic mutations of the main polygenic autoinflammatory syndromes associated with HS. Legend: PASH: Pyoderma gangrenosum, acne, hidradenitis suppurativa; PAPASH: Pyogenic arthritis, Pyoderma gangrenosum, acne, hidradenitis suppurativa; PASS: Pyoderma gangrenosum, acne, hidradenitis suppurativa, ankylosing spondylitis; PsAPASH: Psoriatic arthritis, pyoderma gangrenosum, acne hidradenitis suppurativa; PsAPSASH: Pustular psoriasis, arthritis, pyoderma gangrenosum, synovitis, acne, hidradenitis suppurativa; SAPHO: Synovitis, acne, pustulosis, hyperostosis, osteitis: IL: Interleukin.
